# Case of Extraordinary Constipation of the Bowels Relieved by Medicine

**Published:** 1830-01-01

**Authors:** 


### XVII.

### BATH UNITED HOSPITAL.

[Mr. BURY Reporter.]

### EXTRAORDINARY CONSTIPATION OF THE BOWELS.

Perhaps no instance of more protracted constipation than the subjoined is on record; but the pleasing satisfaction with which the case has terminated under moderate measures steadily pursued, appears more deserving of publication than the relation of the unusual facts themselves. The case was treated by Dr. Barlow, whose kindness has supplied the writer with the circumstantial account of the earlier history and more important particulars, which is principally derived from his hospital journal, and the letters of the medical gentlemen in the country who had the care of the patient before she came to Bath.

Charlotte Councel, æt. 24, applied at the United Hospital of this city, Sept. 6th, 1828, having had no stool whatever for thirteen weeks.

It appears from her own account and the description of her case given by the practitioners who have attended her heretofore, that she has always been of a costive habit, which has, however, yielded to moderate aperients till within the last year. Prior to the time just specified, she had been in the habit of allowing many days to elapse without any motion from the bowels, and this, too, unattended by any very perceptible ill consequences at the time. In December, 1826, she was attacked with inflammation of the lungs, which was removed by ordinary remedies; and at that time there was no apparent obstruction in the intestinal tube. Soon after her recovery from this attack, a circumscribed swelling was observed in the abdomen in the situation of the cæcum, accompanied with bearing-down sensations and slight redness of the integuments. Dropsy was apprehended, but the enlargement subsided after the application of leeches and a blister, and she continued tolerably well for some months. There now came on a great disposition to indolence, with unwillingness to take exercise, so that she was accustomed to sit all day on her chair, from which nothing could induce her to move. The action of the bowels became more irregular (intervals of 8 or 10 days sometimes occurring between each evacuation), and proportionally less influenced by medicine.

On the 10th December, 1827, the patient consulted Dr. Gingell, of Thornbury, where she was then resident, having passed one motion on that day, after complete constipation for five weeks.

Jan. 27th, 1828, a second stool was discharged, the patient having taken salts and senna, in moderate doses, twice a week during the intervening time (a period of seven weeks), agreeably to Dr. G.'s directions. At this time her skin was in a state of constant perspiration, which was occasionally very profuse; her urine was highly-coloured and copious. There was no pain, but frequent uneasiness, felt in the bowels; no enlargement could be discovered in any part of the abdomen, her tongue was clean and moist; pulse 80, soft and regular. She had latterly eaten no animal food, and taken only debilitating fluids, such as milk and water, in small quantities.

A third stool was passed March 30th, after constipation for 9 weeks, consisting of hard and dry scybala, resembling burnt coal in external character, which appearance, we believe, the two previous motions also presented. Calomel and colocynth had been prescribed twice a week, with a mixture containing magn. sulphas; but these were taken very irregularly, sometimes omitted for a fortnight. Some very dark discoloration was now observed in the right hypochondrium, which part was full and hard, and afforded considerable pain and soreness on pressure. Relief was derived from the use of fomentations; and injections of water gruel were given, but no feculent matter was brought away by them.

Another small evacuation (the fourth) was produced on June 8th, an interval of ten weeks, similar to shoe-maker's wax in colour and consistence. It was composed of hard, round scybala, and other pieces of natural figure, an inch in length, and the size of the finger. A few days previous to this date, she took croton oil and cathartic extract; and a tobacco enema was administered, which was followed by violent vomiting of a dark and offensive fluid, like to walnut-catsup, and thought to be stercoraceous. This effect was supposed to have arisen from the tobacco, as she has not vomited since.

No evacuation from the bowels has taken place subsequently to the latter date, a period not less than 13 weeks. Separate combinations of calomel, jalap, and salts, and of calomel, gamboge, and aloes, have been given during the above time in doses progressively increased. The extr. elaterii from half a grain to four grains, and tiglii oleum, in four-minim doses, have been also exhibited, but without effect. The croton oil produced very violent pain of abdomen, profuse perspiration, and coldness of the extremities, each time it was administered, and was, therefore, not persevered with. The patient is emaciated and pale, but her aspect does not indicate the existence of either severe disease or great functional disorder. She has no fever—the catamenia have always been regular, sometimes painful—the pulse is quick—tongue whitish—little appetite or thirst—skin moist—urine high coloured, but not deficient in quantity. There is no general fulness of the abdomen, which feels naturally soft. A distinct swelling, hard and firm to the touch, and causing pain when pressed, is still perceptible in the situation of the head of colon, which we may infer to have existed since March. She has lived of late on bread and milk, the amount of which in the day has not exceeded eight or ten ozs. The following medicines were prescribed.

℞. Ext. Colocynth. c. ʒj. Olei croton. gtt. iij. M. ft. pilulæ duodecim. Capt. j. 4tis horis, c. mist. sequent. ℥j.

℞. Magnes. Sulphat. ℥j. Infus. ros. comp. Oss. M. Enemata ope syringis nocte maneq.

Sept. 10th. Has taken her pills and mixture regularly and had four injections—no feculent discharge—the injections, which are of soap and water, are generally retained. Has not had sickness or vomiting—takes very little food—pulse 96—tongue moist and clean—occasional pains of abdomen. Pergat. c. medicamentis. Liniment. sapon. co. abdomini infricandum.

Sept. 12th. Ext. colocynth. co. gr. iv. Hydr. submur. gr. j. Ft. pil. 6tis horis sumenda. Contr. mist. et enemata.

13th. Medicines taken regularly; injections also used with only a few interruptions—no stool—no sickness or vomiting. States that she feels better, and thinks the bowels seem disposed to act. Pergat.

17th. One feculent stool at length obtained, after more than 14 weeks' perfect constipation. It was voided not without a good deal of pain and was quite hard and solid. Intermitt. remed. hodiè; sed cras repet. pil., mist. et enemata.

25th. Mouth affected—no stool since 17th. Six ounces of blood were taken from the arm, it being supposed that some spasmodic action in the bowels might assist in retaining the fæces—no buff. Interm. pil. et mistura. Habeat pulv. jalapæ co. (pp. Ed.) ℈j. ter die. Contin. enemat. et liniment.

Oct. 3d. Pulv. jal. comp. ʒss. ter die. Cont. cætera.

8th. A small stool this morning of a similar kind to the last. Has used the Cross-bath twice—Pergat.

25th. Another small stool yesterday.

℞. Ext. col. comp. ʒj. Ol. crotonis, gtt. iv. Ext. hyosciami, gr. xvj. M. ft. pil. xij. Capiat duas ter indies, cum mixt. seq. ℥j.

℞. Magnesiæ sulph. ℥j. Infus. ros. co. Oss. Tinct. jalapæ, ℥j. M.

Nov. 1st. Another small stool. Pergat.

7th. A stool of five scybala, round, and passed with considerable pain, and as well as the former nearly black in colour. Perg.

19th. A stool also of five scybala, but less dark—vertigo and cephalalgia. Cont. medic. Vesicat. ij. ponè aures.

Nov. 25th. A pulpy stool of some consistence. Perg.

Dec. 3. A stool of about six small balls.

℞. Extr. col. co. ℈ivss. Cambogiæ, gr. x. Tiglii olei, ♏v. M. ft. pil. xx. Sumt. ij. ter die c. misturâ.

13th. A free stool this morning—some scybala and some pulpy fæces. Rep. pil. ut prescript. Oct 25. Cont. cætera.

15th. A stool of two scybala.

17th. Habeat pil. præscript. die 3.

21st. A scybalous stool.

23d. A pulpy motion.

27th. A large pulpy stool, making three within 10 days.

30th. Stool consisting of scybala, scanty and dark.

Jan. 6th, 1829. A stool, scanty but pulpy.

8th. A stool, composed of scybala.

13th. A stool, pulpy and more copious.

15th. A stool of same character; and on 22d also a full, pulpy motion.

Feb. 14th. No account has been kept since last report of the number of stools, which have been more frequent and are much improved in character, under the continued use of purgatives, in nearly as large quantity.

The patient to-day left Bath for the country, and did not return till June 23d, looking much better and having gained flesh. At her departure, we believe she had lost every appearance of health, as was to be expected; but the constitution, throughout the treatment, bore up almost marvellously against her ailments.

She brought with her the following report of the motions from her bowels during her absence. Feb. 22d, a stool; 28th, a stool. Between these two dates she suffered much in her head, being at times deprived of her senses. The bowels did not act again before March 22d; but on the 26th, 28th, and 30th, she passed a motion each day. During the month of April she had stools on the 1st, 2d, 5th, 6th, 8th, 11th, 14th, 17th, 22d, 24th, 25th, and 26th. On the following days in May, viz. 1st, 2d, 3d, 4th, 8th, 10th, 12th, 13th, 19th, 21st, 25th, 27th and 28th.

A motion was passed June 2d, and from that day up to the 21st she had a daily evacuation.

June 27th. She applied at the hospital this day, as the bowels still require the stimulus of purgatives. She states that at the early part of her absence, strong cathartic doses have been requisite to open the bowels, but during the latter part much less powerful ones have sufficed. She has gained much in flesh, and her general health and appearance are greatly improved—pulse frequent but soft—tongue furred and white—catamenia scanty but regular.

Ordered Pil. aloës comp. gr. x. o. n. Magnesiæ sulph. ʒj. Inf. ros. co. ℥j. Ft. h. ter quotidie sumendus.

The patient attended throughout July, the bowels being at times much disposed to costiveness, which, however, exceeded not three or four days. There was a constant flushing of both cheeks after she had been in conversation a few minutes. The medicines above directed were continued through the month, about the middle of which some feverishness and pains of head were removed by a moderate blood-letting.

During August, her reports were more favourable than at any foregoing period. She got a natural pulpy stool every second day, of proper colour, and discharged without any of her old distressing uneasiness of abdomen. Her appetite was considered good; she had no thirst, and had increased in embonpoint. The medicines were necessary, though in smaller doses.

Sept. 8th. Has two healthy and easy motions in three days, and may be said to ail nothing, with the exception of slight recurrent pains of head and side. Small doses of purgative medicine are still continued, and she says she is confident they cannot be yet dispensed with.

LIST OF STOOLS.

No. 1. Dec. 10, 1827, after constipation of 5 weeks.—2. Jan. 27, 1828, ditto 7.—3 March 30, ditto 9.—4. June 8, ditto 10.—5. Sept. 17, ditto 14 *Five stools in 45 weeks.*

No. 6. Oct. 8, after constipation of 3 weeks.—7. Oct. 25, ditto 17 days. *Two motions in month of October.*

No. 8. Nov. 1, after constipation of 7 days.—9. Nov. 7, ditto 6.—10. Nov. 19, ditto 12.—11. Nov. 25, ditto 6. *Four stools in month of November.*

No. 12. Dec. 3, after constipation of 8 days.—13. Dec. 13, ditto 10.—14. Dec. 15, ditto 2.—15. Dec. 17, ditto 2.—16. Dec. 21, ditto 4.—17. Dec. 23, ditto 2.—18. Dec. 27, ditto 4.—19. Dec. 30, ditto 3. *Eight stools in Dec.*

No. 20. Jan. 6, 1829, after constipation of 7 days.—21. Jan. 8, ditto 2.—22. Jan. 13, ditto 5.—23. Jan. 15, ditto 2.—24. Jan. 22, ditto 7. *Five stools in 23 days.*

---

XVIII.

